# Comparison of in vitro and in vivo repellency bioassay methods for *Ixodes* *scapularis* nymphs

**DOI:** 10.1186/s13071-023-05845-7

**Published:** 2023-07-10

**Authors:** James C. Burtis, Shelby L. Ford, Christina M. Parise, Erik Foster, Rebecca J. Eisen, Lars Eisen

**Affiliations:** Division of Vector-Borne Diseases, National Center for Emerging and Zoonotic Infectious Diseases, Centers for Disease Control and Prevention, Fort Collins, CO 80521 USA

**Keywords:** *Ixodesscapularis*, In vitro bioassays, In vivo bioassays, Repellency, DEET, Peppermint oil, Rosemary oil, Geographic origin

## Abstract

**Background:**

Numerous bioassay methods have been used to test the efficacy of repellents for ticks, but the comparability of results across different methods has only been evaluated in a single study. Of particular interest are comparisons between bioassays that use artificial containers (in vitro) with those conducted on a human subject (in vivo) for efficacy testing of new potential unregistered active ingredients, which most commonly use in vitro methods.

**Methods:**

We compared four different bioassay methods and evaluated three ingredients (DEET [N,N-Diethyl-meta-toluamide], peppermint oil and rosemary oil) and a negative control (ethanol) over a 6-h period. Two of the methods tested were in vivo bioassay methods in which the active ingredient was applied to human skin (finger and forearm bioassays), and the other two methods were in vitro methods using artificial containers (jar and petri dish bioassays). All four bioassays were conducted using *Ixodes* *scapularis* nymphs. We compared the results using nymphs from two different tick colonies that were derived from *I.* *scapularis* collected in the US states of Connecticut and Rhode Island (northern origin) and Oklahoma (southern origin), expecting that ticks of different origin would display differences in host-seeking behavior.

**Results:**

The results between bioassay methods did not differ significantly, even when comparing those that provide the stimulus of human skin with those that do not. We also found that tick colony source can impact the outcome of repellency bioassays due to differences in movement speed; behavioral differences were incorporated into the assay screening. DEET effectively repelled nymphs for the full 6-h duration of the study. Peppermint oil showed a similar repellent efficacy to DEET during the first hour, but it decreased sharply afterwards. Rosemary oil did not effectively repel nymphs across any of the time points.

**Conclusions:**

The repellency results did not differ significantly between the four bioassay methods tested. The results also highlight the need to consider the geographic origin of ticks used in repellency bioassays in addition to species and life stage. Finally, our results indicate a limited repellent efficacy of the two essential oils tested, which highlights the need for further studies on the duration of repellency for similar botanically derived active ingredients and for evaluation of formulated products.

**Graphical Abstract:**

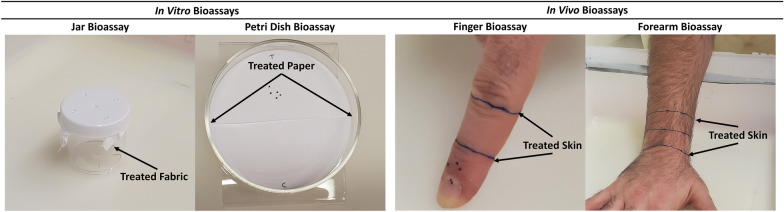

## Background

Ticks and tick-borne diseases pose a major threat to human health in the USA. The blacklegged tick (*Ixodes* *scapularis*) is the primary vector of multiple human pathogens in the eastern USA, including *Borrelia* *burgdorferi* sensu stricto, *B.* *mayonii* (Lyme disease), *B.* *miyamotoi* (hard tick relapsing fever), *Anaplasma* *phagocytophilum* (anaplasmosis), *Babesia* *microti* (babesiosis), *Ehrlichia* *muris* *eauclairensis* (ehrlichiosis) and Powassan virus (Powassan virus disease) [1]. Safe, effective and accessible methods to deliver broad-scale or area-wide control of *I.* *scapularis* are currently lacking [2]. Therefore, the best defense against *I.* *scapularis*-borne pathogens is to reduce tick-human contact rates through tick habitat avoidance and the use of personal protection methods, including tick repellents [3]. There are a number of US Environmental Protection Agency (EPA)-registered repellents on the market that are effective against *I.* *scapularis*, including, but not limited to, those containing N,N-Diethyl-meta-toluamide (DEET), picaridin, IR3535, oil of lemon eucalyptus, para-menthane-diol or 2-undecanone as active ingredients [4, 5]. New repellent active ingredients are also under development, and some existing repellent active ingredients and formulations have not been evaluated.

Repellency bioassays are commonly used to evaluate the efficacy of repellents for use against ticks. There are a variety of bioassay methods that target different tick species and life stages, including both in vivo (using a live subject) and in vitro (not using a live subject) methods. However, there is limited standardization between methods, as concentrations of the targeted active ingredients and volume per treatment area may vary [6, 7]. It is therefore difficult to compare the results between bioassay methods, particularly between in vivo and in vitro bioassays where stimuli from a live subject may affect the results of the former. The EPA recommends the in vivo forearm bioassay as the primary method to evaluate tick repellents, but this may not work across all species and life stages due to differences in mobility. EPA guidelines do suggest a consistent concentration (20% solution) and volume per unit area (1 ml per 600 cm^2^) for the forearm bioassay [8], and these provide good baseline application rate targets for the comparison of results across bioassay methods.

In the development of mosquito and tick repellents, the EPA classifies some chemical compounds as ‘exempt’ from registration, referred to as ‘25(b) exempt,’ as they have been determined to minimally impact human health [9]. When these ‘25(b) exempt’ compounds are used as the active ingredient for commercial repellent formulations, no efficacy testing is required. Many botanically derived essential oils are included in this category (e.g. peppermint oil and rosemary oil). Efficacy data for ‘25(b) exempt’ active ingredients and commercial products against ticks come from research studies and are available only for a small portion of the products on the market. This research has been conducted using a variety of bioassay methods [6, 10–12], making it difficult to compare the efficacy of active ingredients between studies.

Given the wide variety of bioassay methods used for efficacy evaluations, as well as the practical benefits of using in vitro methods for initial screening of novel active ingredients, it is important to determine whether results differ between bioassay methods when tick life stage, age, rearing conditions and the application rate of the active ingredients are standardized. In the present study, we tested two common in vivo methods (finger bioassay and forearm bioassay) and two in vitro methods (jar bioassay and petri dish bioassay) using *I.* *scapularis* nymphs. Nymphs were used as they represent the life stage accounting for most human Lyme disease, anaplasmosis and babesiosis infections [13–15] and are easier to rear in large numbers, allowing us to use naïve nymphs for each time point when determining the duration of repellency. The study included a negative control (ethanol) and three repellent treatment groups: positive control (DEET) and two ‘25(b) exempt’ oils (peppermint oil and rosemary oil). To determine whether colony origin affected the bioassay results, repellency outcomes were compared using nymphs from two separate laboratory colonies that were expected to yield differences in nymphal questing behavior: colony ticks originated from the US states of Connecticut and Rhode Island (USA) and colony ticks from the US state of Oklahoma (USA). Under natural conditions, nymphs of northern origin tend to display more aggressive host-seeking behavior compared with nymphs derived from the south [16]. We sought to determine if repellency was overestimated when using the in vitro bioassays, due to the lack of a human attractant, and to assess the performance of the different bioassays when used with the DEET gold standard repellent versus potentially less repellent ‘25(b) exempt’ active ingredients.

## Methods

### Tick rearing

We used *I.* *scapularis* colonies of two origins in our bioassays, one derived from specimens collected in Connecticut and Rhode Island (northern origin), which is maintained at the Centers for Disease Control and Prevention, Division of Vector-Borne Diseases in Fort Collins, Colorado (CDC colony hereafter), and one derived from specimens collected in Oklahoma (southern origin) and maintained at Oklahoma State University, Stillwater, Oklahoma (OSU colony hereafter). The OSU colony was started in 1991, while the CDC colony was started in 2003. Both colonies are intermittently refreshed with ticks from the field, either from Connecticut and Rhode Island (CDC) or from Oklahoma (OSU), to maintain genetic diversity. At OSU, all tick life stages are fed on sheep and rabbits. OSU colony larvae shipped to CDC were fed on CD1 mice (Charles River Laboratories, Wilmington, MA, USA) and allowed to molt into nymphs for use in the bioassays. In the CDC colony, adults were fed on New Zealand white rabbits and larvae on CD1 mice to produce nymphs for the bioassays. Fed larval ticks were cleaned using water and filter paper and maintained at 24 °C in desiccators with a potassium sulfate solution (120 g/l) to maintain high relative humidity until the nymphs molted. Nymphs used in repellency bioassays were between 2- and 4-weeks post-molt. Animal use and experimental procedures were in accordance with approved protocols on file with the Centers for Disease Control and Prevention, Division of Vector-Borne Diseases Animal Care and Use Committee.

### Repellency bioassays

Four repellency bioassay methods were evaluated in this study, of which two were in vivo methods and conducted on human skin (forearm bioassay and finger bioassay) and two were in vitro methods and conducted within artificial containers (jar and vertically oriented petri dish bioassays). The finger bioassay [17] was originally designed for use with nymphs. The jar bioassay is a modification of the Falcon vial bioassay, using a wider container, and was also originally designed for use with nymphal ticks [18, 19]. The forearm and vertical petri dish bioassays were designed for use with adult ticks [8, 20] and rely on the proclivity of adult *I.* *scapularis* to quest upwards. Nymphs do not climb as predictably or quickly as adults, and nymphal behavior is expected to differ based on the origin of the nymphs. Therefore, we modified the forearm and vertical petri dish assays so that nymphs were placed on the treated area, rather than below it. Repellency was recorded when they crawled off the treated area prior to a time point determined as described in the following subsections. For the finger bioassay, the treated area was small relative to the forearm bioassay, so nymphs crawled upwards through it reliably. Nymphs also tended to spread out of the small, treated area of the finger rapidly, making it difficult to place five of them within the treated area before at least one had already crawled away.

We standardized the volume of the test solution (negative control: ethanol [ETOH]; repellent active ingredients: DEET [Chem Service Inc., West Chester, PA, SKU: –12618], peppermint oil [Sigma-Aldrich, St. Louis, MO, SKU: W284815] or rosemary oil [Sigma-Aldrich, SKU: W299200] dissolved in ETOH) applied for each bioassay to 1 ml per 600 cm^2^ of treated area in accordance with guidance from the EPA [8], and a 20% solution was used for each of the three active ingredients. We conducted pre-trials with no test solution applied to the test surface prior to each trial to ensure that the test nymphs displayed normal behavior and mobility (i.e. crawling upwards through the test area for the finger bioassay or crawling off the test surface for the other three bioassays). Nymphs were tested in sets of five in the pre-trials, and if > 50% of nymphs in a set did not exhibit normal behavior, the set was discarded and another five ticks were tested. Sets of nymphs performing as expected in the pre-trials were then used in the trials with test solutions. We tested a total of six rounds of five nymphs at each of the seven time points (0–6 h) for each bioassay, active ingredient and tick colony. Therefore, a total of 210 nymphs were used to test each bioassay method and repellent combination. Each time point used separate sets of naïve ticks so that the observations were independent. Positive (DEET) and negative (ETOH) control bioassays were conducted with nymphs from both the OSU and CDC colonies to explore the effect of behavioral differences on the results. Based on their higher level of activity, only nymphs from the CDC colony were used to evaluate the efficacy of peppermint and rosemary oil as repellents.

#### Jar bioassay

**Fig. 1:**
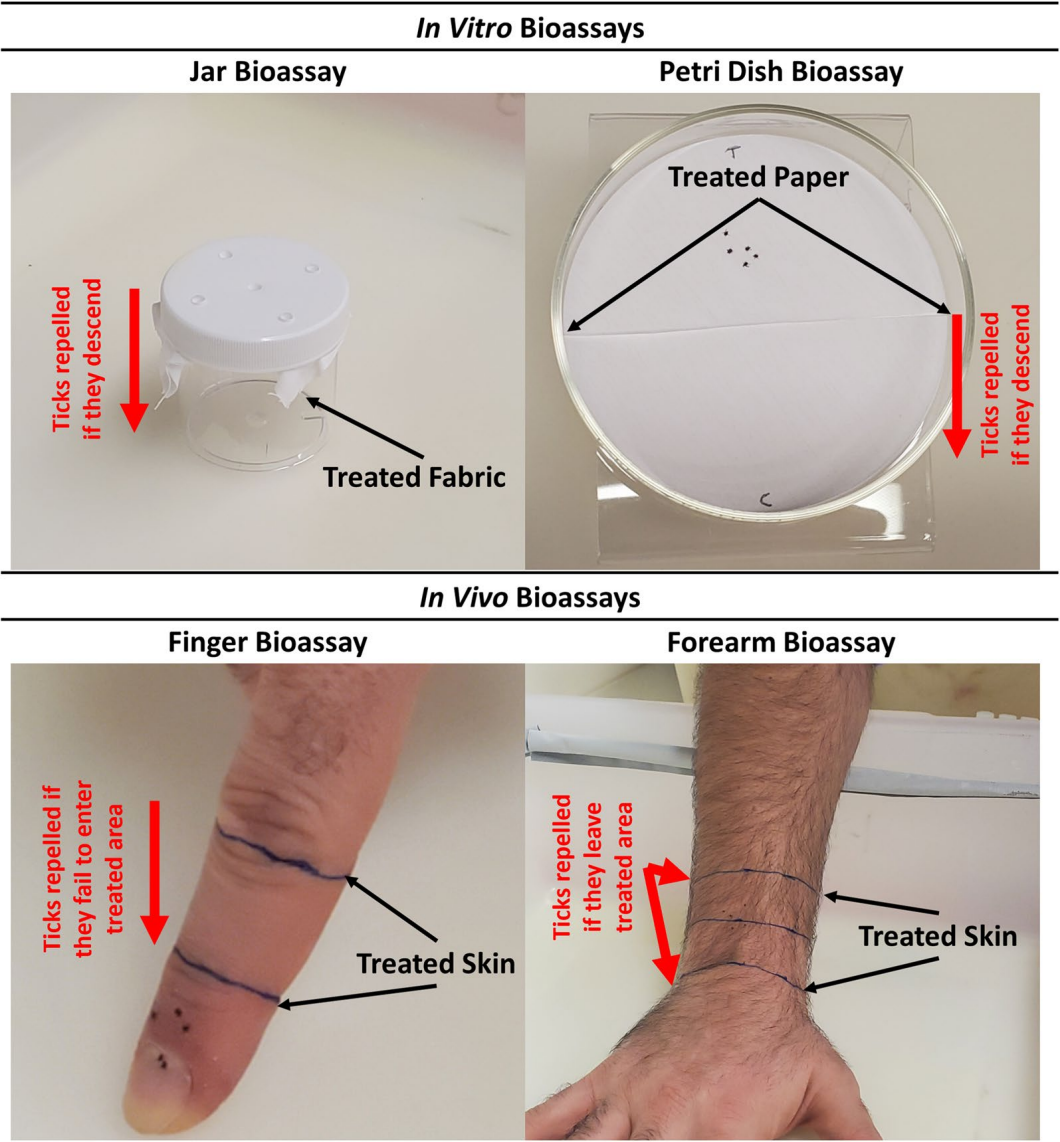
Setup for the four bioassay methods used to assess repellency toward *Ixodes* *scapularis* nymphs. For the finger and forearm bioassays, the area between the arrows was treated; for the jar bioassay, the entire piece of fabric was treated; for the petri dish bioassay, the top half of the filter paper was treated. For the jar, vertical petri dish and forearm bioassays, ticks are placed within the treated area and the numbers that remain inside versus crawl out of the treated area are recorded at the repellency assessment time point. Nymphs in the finger bioassay are introduced below the treated area and the numbers remaining below versus crawling onto or through the treated area are recorded

The jar bioassay was an in vitro bioassay that we conducted inside a 90-ml-capacity plastic jar (model S-17034; Uline, Pleasant Prairie, WI, USA) with five holes punched in the cap (Fig. 1). A piece of 8 × 8-cm white cotton fabric was treated with 107 µl of the test solution (i.e. ETOH, or a 20% solution of DEET, peppermint oil or rosemary oil). The fabric was allowed to dry under a chemical hood for 20 min. Once the fabric was treated and dried, we placed five nymphs on it, and the fabric was placed on the underside of the jar’s cap; the inverted jar was then affixed to the cap, with the fabric secured by the threads of the jar and cap. The jar was then placed right side up so that the nymphs were upside-down at the top of the jar on the fabric. Ticks were placed on the top of the jar to force them to move downward rather than upward and alter their normal tendency to remain attached to an untreated surface.

For the jar bioassay, the time to repellency was determined by first applying nymphs to negative control assays (using ETOH only) and recording the amount of time it took for the first nymph to crawl off the ETOH-treated surface. We then applied nymphs to positive control assays (using 20% DEET dissolved in ETOH) and recorded the time when 100% of ticks had crawled off the DEET-treated surface. This was repeated with 20 sets of five nymphs (10 sets with DEET as positive control and 10 sets with ETOH as negative control) for each of the two tick colonies. The repellency assessment time point is an intermediate time between these two time points rounded to the nearest 5 s. When possible, time points were matched between bioassay methods or colonies (Table 1). Nymphs of different origin showed variable levels of activity, and thus the time point to assess repellency was 75 s for CDC nymphs and 130 s for those from the OSU colony. Nymphs either walked off or dropped off the fabric. We recorded the number of nymphs still on (not repelled) on the treated fabric versus those having moved off of it (repelled) at the repellency assessment time points.

**Table 1:** The data used to determine the repellency assessment time points (in seconds) for each of the four bioassays using *Ixodes* *scapularis* nymphs from two tick colonies

Bioassay	Tick colony^a^	Positive control (DEET)	Negative control (ETOH)	Selected repellency time point
Jar bioassay	CDC	16 s	120 s	75 s
	OSU	119 s	142 s	130 s
Petri dish bioassay	CDC	72 s	97 s	75 s
	OSU	117 s	181 s	130 s
Finger bioassay	CDC	> 600 s	67 s	75 s
	OSU	> 600 s	332 s	340 s
Forearm bioassay	CDC	181 s	201 s	190 s
	OSU	176 s	201 s	190 s

The time in the ‘positive control’ column represents the longest time for 100% of nymphs to be repelled in a positive control DEET treatment. Times in the ‘negative control’ column represent the shortest time that the first nymph was “repelled” in a negative control ETOH treatment. The repellency assessment time point is an intermediate time between the other two time points rounded to the nearest 5 s. When possible, time points were matched between bioassay methods or colonies

*DEET* N,N-Diethyl-meta-toluamide,* ETOH* ethanol

^a^CDC colony was derived from specimens collected in Connecticut and Rhode Island (northern origin) and maintained at the Centers for Disease Control and Prevention, Division of Vector-Borne Diseases in Fort Collins, Colorado. OSU colony was derived from specimens collected in Oklahoma (southern origin) and maintained at Oklahoma State University, Stillwater, Oklahoma

#### Vertical petri dish bioassay

The petri dish bioassay was an in vitro bioassay conducted inside an open petri dish that was tilted at a 45° angle (Fig. 1). A round piece of Whatman filter paper (diameter: 90 mm) was cut in half, with one half being treated (using 53 µl of test solution) and the other left untreated. The treated filter paper was allowed to dry under a chemical hood for 20 min. We then placed the two filter paper halves inside the petri dish, with the treated half at the top, and five nymphs were placed in the middle of the treated half of the filter paper. Ticks were placed on the top half of the petri dish to force them to move downslope rather than upward, again forcing them to move against their normal tendency to ascend.

For the petri dish bioassay, the time to repellency was determined by first applying nymphs to negative control assays (using ETOH only) and recording the amount of time it took for the first nymph to crawl off the ETOH-treated surface. We then applied nymphs to positive control assays (using 20% DEET dissolved in ETOH) and recorded the time when 100% of ticks had crawled off the DEET-treated surface. This was repeated with 20 sets of five nymphs (10 sets with the DEET positive control and 10 with the ETOH negative control) for each of the two tick colonies. The repellency assessment time point is an intermediate time between these two time points rounded to the nearest 5 s. When possible, time points were matched between bioassay methods or colonies (Table 1). The time point for assessing repellency was 75 s for nymphs from the CDC colony and 130 s for nymphs from the OSU colony. We recorded the number of nymphs still on (not repelled) the treated paper versus the number having moved off (repelled) the treated paper at the repellency assessment time points.

#### Finger bioassay

The finger bioassay was an in vivo bioassay, conducted on the index finger of a human subject. The assay is based on the natural inclination of *I.* *scapularis* nymphs to move upward on a vertically positioned human skin surface, such as a finger or an arm. We drew lines at the first and second knuckle (Fig. 1) and treated the area between the two knuckles with 38 µl of test solution. The finger was then allowed to dry under a chemical hood for 20 min. Once treated and dried, the tip of the finger was placed against a table with the finger at a 90° angle. Five nymphs were placed at the nail bed, and they were given the opportunity to climb upwards through the treated area.

To determine the repellency time point for the finger bioassay, we recorded the time when 100% of nymphs had crawled through the ETOH-treated portion of the finger, using 10 sets of five nymphs from each colony. The repellency time point selected was 5 s above the slowest nymph rounded to the nearest 5 s time point. We added the extra 5 s to create a short time buffer in case nymphs occasionally moved slightly slower than those in our initial testing. The time point for assessing repellency was 80 s for nymphs from the CDC colony and 340 s for those from the OSU colony. At the repellency assessment time points, we recorded the number of nymphs that were still below the treated area (repelled) versus those that had entered or crawled through the treated area (not repelled). If a nymph crossed into the treated area, they were not considered to be repelled. Only one person was used as a subject for this experiment to reduce variation in results between participants.

#### Forearm bioassay

The standard in vivo EPA forearm bioassay [8] uses adult *I.* *scapularis* and, similar to the finger bioassay, relies on the inclination of the ticks to crawl upwards on a vertically oriented surface. Nymphs move more slowly and less predictably compared to adults, particularly on a large surface like the forearm. We therefore placed the nymphs directly on the treated portion of the forearm and they were allowed to crawl out of this area. Three lines were drawn on the subject’s forearm, one at the base of the wrist, one 3 cm up from that and a third 6 cm from the base of the wrist (Fig. 1). We treated the entire forearm area (top and bottom) between the first and third lines with 184 µl of test solution. The forearm was then allowed to dry under a chemical hood for 20 min. Once treated and dried, the forearm was held at a 45° angle, and we placed five nymphs on the middle line in the center of the forearm.

For the forearm bioassay, the time to repellency was determined by first applying nymphs to negative control assays (using ETOH only) and recording the amount of time it took for the first nymph to crawl off the ETOH-treated surface. We then applied nymphs to positive control assays (using 20% DEET dissolved in ETOH) and recorded the time when 100% of ticks had crawled off the DEET-treated surface. This was repeated with 20 sets of five nymphs (10 sets with the DEET positive control and 10 with the ETOH negative control) for each of the two tick colonies. The repellency assessment time point is an intermediate time between these two time points rounded to the nearest 5 s. When possible, time points were matched between bioassay methods or colonies. Nymphs from both colonies behaved similarly for the forearm bioassay, likely due to the relatively large distance they needed to move to be considered repelled (Table 1). Therefore, the time point for assessing repellency was 190 s for nymphs from both the CDC and OSU colonies. At the repellency assessment time point, we recorded the number of ticks still inside the treated are versus the number outside of the treated area. As with the finger bioassay, only one person was used as a subject for the forearm bioassay.

### Statistical analyses

Repellency data were analyzed using logistic regressions for proportions coded as generalized linear mixed models using the glm() command in R with the logit link function, weighted by the total number of ticks in each bioassay. The response was a proportional variable where repellency was coded as the number of ticks repelled over the total number of ticks in a single bioassay (i.e. replicate), which was five in all cases. Results were considered significant at α = 0.05. Statistical analyses were conducted using R version 4.2.1 [21].

For analysis of the pre-trial data, ticks were scored as displaying expected behavior or not. In the forearm, vertical petri dish and jar bioassays, expected behavior meant remaining within the test area at the repellency time point; for the finger bioassay, it meant crawling into or through the test area. We used two logistic regressions for proportions (one for the OSU colony and one for the CDC colony) to compare the behavior between ticks applied to an untreated test substrate (untreated filter paper, fabric or skin) relative to that of ticks applied to the test substrate treated with ethanol (negative control). The fixed effects included in this analysis were bioassay method, duration after repellent application (hours 0–6) and treatment (untreated pre-test and ethanol). This analysis was conducted to determine whether use in the bioassays during the pre-trials altered tick behavior in the corresponding trials. The tick behavior data used for this analysis were therefore not corrected using the Abbott formula described below.

Repellency data for all remaining analyses were corrected against their associated pre-trial data using Abbott’s corrected mortality formula, bounded by 0% and 100% [22]:

$${\text{Abbott Corrected Repellency}}=\frac{\% {\text{Repelled in Trial}}-\% {\text{Repelled in Pretrial }}}{100- \% {\text{Repelled in Pretrial}}} \times 100.$$

Another logistic regression for proportions was used to compare the ethanol treatment (negative control) and DEET treatment (positive control) bioassay results between the CDC and OSU tick colonies. In this analysis, the bioassay method, duration after repellent application and tick colony origin (CDC and OSU) were included as fixed effects. This analysis allowed us to determine whether the results of the repellency bioassay methods differed between the two colonies once appropriate repellency time points were determined. A final logistic regression for proportions was conducted using only the bioassay results for the CDC colony comparing the repellent efficacy of the rosemary and peppermint oils with that of the positive (DEET) and negative (ethanol) controls. For this analysis, the fixed effects included bioassay method, duration after repellent application and treatment (ethanol, DEET, rosemary oil, and peppermint oil). This allowed us to determine the efficacy of the two ‘25(b) exempt’ natural active ingredients to act as repellents over a 6-h period.

## Results

### Pre-trial data

Our first two analyses compared the proportion of nymphs displaying expected behavior when applied to the test substrate without application of ethanol to the negative control where ethanol was applied; separate analyses were run for each colony. For nymphs from both the CDC and OSU colonies at each time point after application and across all methods, the application of ethanol resulted in no significant differences in behavior compared with ticks applied to an untreated test substrate (Table 2).

**Table 2:** The average percentage of *Ixodes* *scapularis* nymphs from the CDC and OSU colonies not exhibiting expected behavior for each of the treatment groups, bioassay methods and time points in the pre-trials for the negative controls

	Percent not exhibiting expected behavior	*df*	Deviance residual	*P*-value
CDC colony				
Treatment group				
Pre-trial (untreated surface)	18.0 (15.5*–*20.7) [151/840]	1325	0.04	0.841
Negative control (ETOH treated surface)	17.1 (14.8*–*19.8) [144/840]			
Bioassay method				
Finger bioassay	16.2 (13.0*–*20.0) [68/420]	3326	0.61	0.894
Forearm bioassay	19.0 (15.6*–*23.1) [80/420]			
Jar bioassay	32.6 (28.3*–*37.2) [137/420]			
Petri dish bioassay	15.7 (12.5*–*19.5) [66/420]			
Timepoint in hours after treatment				
0	19.6 (15.1*–*25.1) [47/240]	6329	0.89	0.989
1	18.3 (14.0*–*23.7) [44/240]			
2	18.3 (14.0*–*23.7) [44/240]			
3	20.0 (15.4*–*25.5) [28/240]			
4	16.7 (12.5*–*21.8) [40/240]			
5	14.2 (10.3*–*19.1) [34/240]			
6	15.8 (11.8*–*21.0) [38/240]			
OSU colony				
Treatment group				
Pre-trial (untreated surface)	11.3 (9.3*–*13.6) [95/840]	1325	0.01	0.945
Negative control (ETOH treated surface)	11.5 (9.6*–*13.9) [95/840]			
Bioassay method				
Finger bioassay	14.0 (11.0*–*17.7) [59/420]	3326	7.32	0.062
Forearm bioassay	4.0 (2.5*–*6.4) [17/420]			
Jar bioassay	20.0 (16.5*–*24.1) [84/420]			
Petri dish bioassay	14.3 (11.3*–*18.0) [60/420]			
Timepoint in hours after treatment				
0	13.3 (9.6*–*18.2) [32/240]	6329	1.27	0.973
1	12.5 (8.9*–*17.3) [30/240]			
2	10.0 (6.8*–*14.4) [24/240]			
3	9.2 (6.1*–*13.4) [22/240]			
4	9.2 (6.1*–*13.4) [22/240]			
5	14.6 (10.7*–*19.6) [35/240]			
6	11.3 (7.8*–*15.9) [27/240]			

^a^Expected behavior consisted of, for example, crawling upwards through the test area for the finger bioassay or crawling off the test surface for the other three bioassays. The values in parentheses are the 95% Wilson scores. The values in square brackets are the proportion of ticks discarded for each category

^b^The statistical information relates to the results of the logistic regressions for proportions

The number of nymphs that were discarded across all pre-trials varied by colony, and since there were no OSU trials for peppermint or rosemary oil, we compared the total number of ticks discarded during the pre-trials for the negative and positive controls. In this case, ticks were only discarded if > 50% (> 2 ticks) per replicate did not display the expected behavior. For the CDC colony, 8% [57/720] of nymphs were discarded during finger bioassay pre-trials, 6% [43/720] of nymphs during the forearm bioassays, 7% [50/720] of nymphs during the jar bioassays and 3% of nymphs [22/720] during the petri dish bioassays. For the OSU colony, 19% [137/720] of nymphs were discarded during finger bioassay pre-trials, 5% [36/720] of nymphs during the forearm bioassays, 18% of nymphs [130/720] during the jar bioassays and 7% [50/720] of nymphs during the petri dish bioassays.

We compared our uncorrected (no Abbott correction applied) negative control results against the results of the associated pre-trials to determine whether contact with the bioassay apparatus or human skin had altered tick behavior. We also wanted to determine whether the presence of ethanol in the negative control elicited behavioral effects. The results did not differ for either the CDC (*df* = 1325, deviance residual = 0.04, *P* = 0.841) or OSU (*df* = 1325, deviance residual = 0.01, *P* = 0.973) colonies. The results also did not vary by bioassay (CDC: *df* = 3326, deviance residual = 0.610, *P* = 0.894; OSU: *df* = 3326, deviance residual = 7.32, *P* = 0.062) or time point after application (CDC: *df* = 6329, deviance residual = 0.89, *P* = 0.898; OSU: *df* = 6329, deviance residual = 1.27, *P* = 0.973).

### Colony comparisons for positive control (DEET) and negative control (ethanol)

**Fig. 2:**
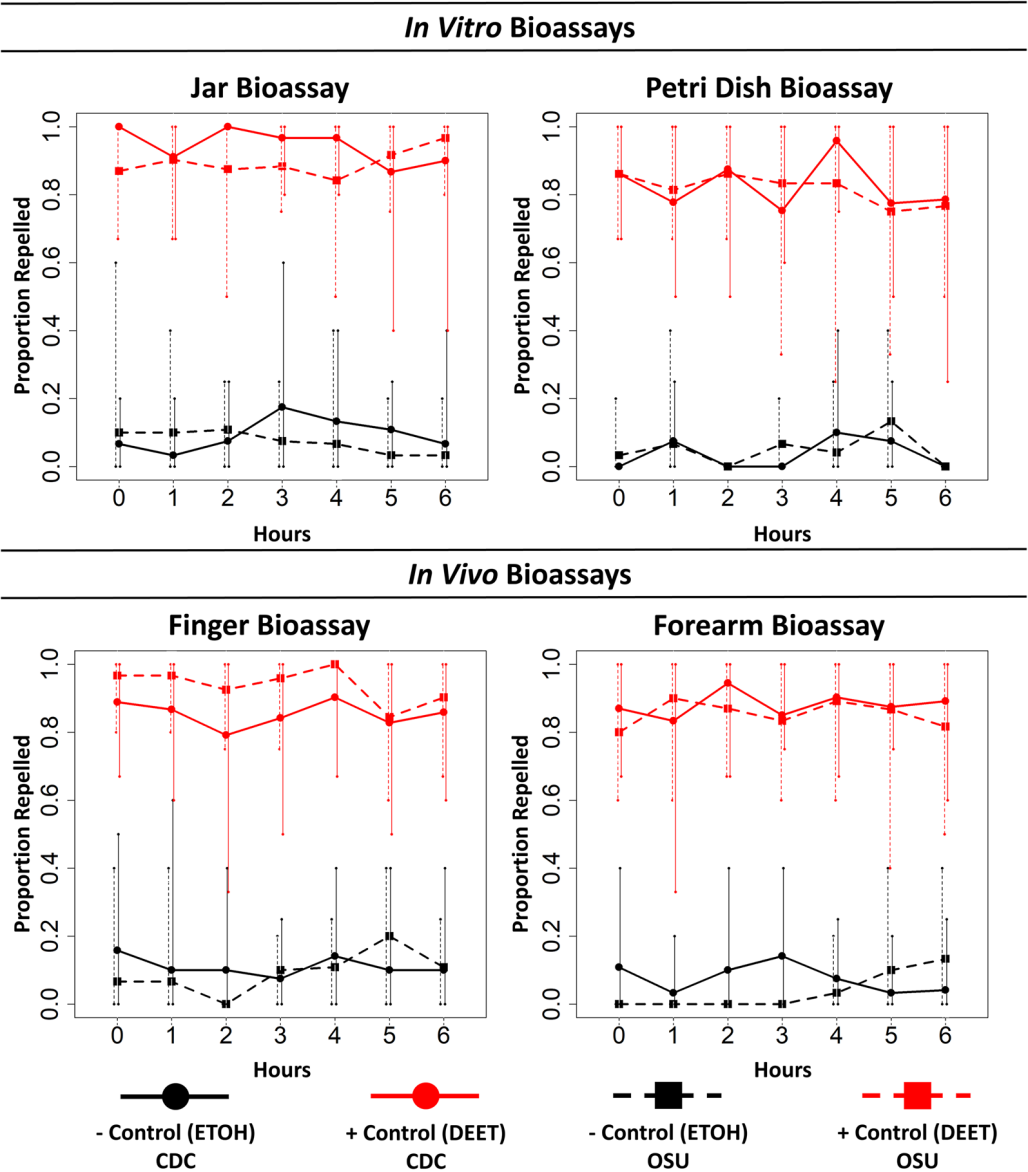
Plots showing the point estimates and range (minimum and maximum across replicates) for each time point after treatment for the negative (ETOH) and positive (DEET) controls, shown in black and red, respectively. The solid lines and filled circle points show repellency for the CDC colony, and the dashed lines and filled squares show repellency for the OSU colony. The proportion repelled for the negative control (ETOH) group was a measure of the portion of nymphs that did not exhibit the expected behavior, rather than true repellency. CDC, Colony derived from specimens collected in Connecticut and Rhode Island and maintained at the Centers for Disease Control and Prevention, Division of Vector-Borne Diseases (Fort Collins, CO, USA); DEET, N,N-Diethyl-meta-toluamide; ETOH, ethanol; OSU, colony derived from specimens collected in Oklahoma and maintained at Oklahoma State University (Stillwater, OK)

Across each of the bioassays and time points after treatment, the proportion of nymphs repelled by DEET or ethanol was similar between nymphs derived from the CDC and OSU colonies (*df* = 1324, deviance residual = 0.067, *P* = 0.796). The proportion of ticks repelled was greater for our positive control (DEET) than for the negative control (ethanol) (*df* = 1325, deviance residual = 296.1, *P* < 0.001) (Fig. 2). Nymphs in the negative control (ethanol) group were not truly ‘repelled,’ but rather failed to exhibit the expected behavior. Despite this, for simplicity, we refer to nymphs which did not display the expected behavior as ‘repelled’ in the text hereafter, as well as in our figures. The Abbott correction is bounded by 0 on the low end. Therefore, the average ‘repellency' in the negative control is slightly higher than expected since any negative numbers were increased to 0. Across all bioassay methods and time points after treatment, for the CDC colony, 8% [58/720] were repelled in the negative control (ethanol) and 88% [634/720] were repelled in the positive control (DEET). For the OSU colony, 6% [43/720] were repelled in the negative control (ethanol) and 88% [634/720] were repelled in the positive control (DEET). The results also did not differ significantly between time points after treatment (*df* = 6329, deviance residual = 0.517, *P* = 0.998) or across bioassay methods (*df* = 3326, deviance residual = 1.02, *P* = 0.797).

### Natural repellents

**Fig. 3:**
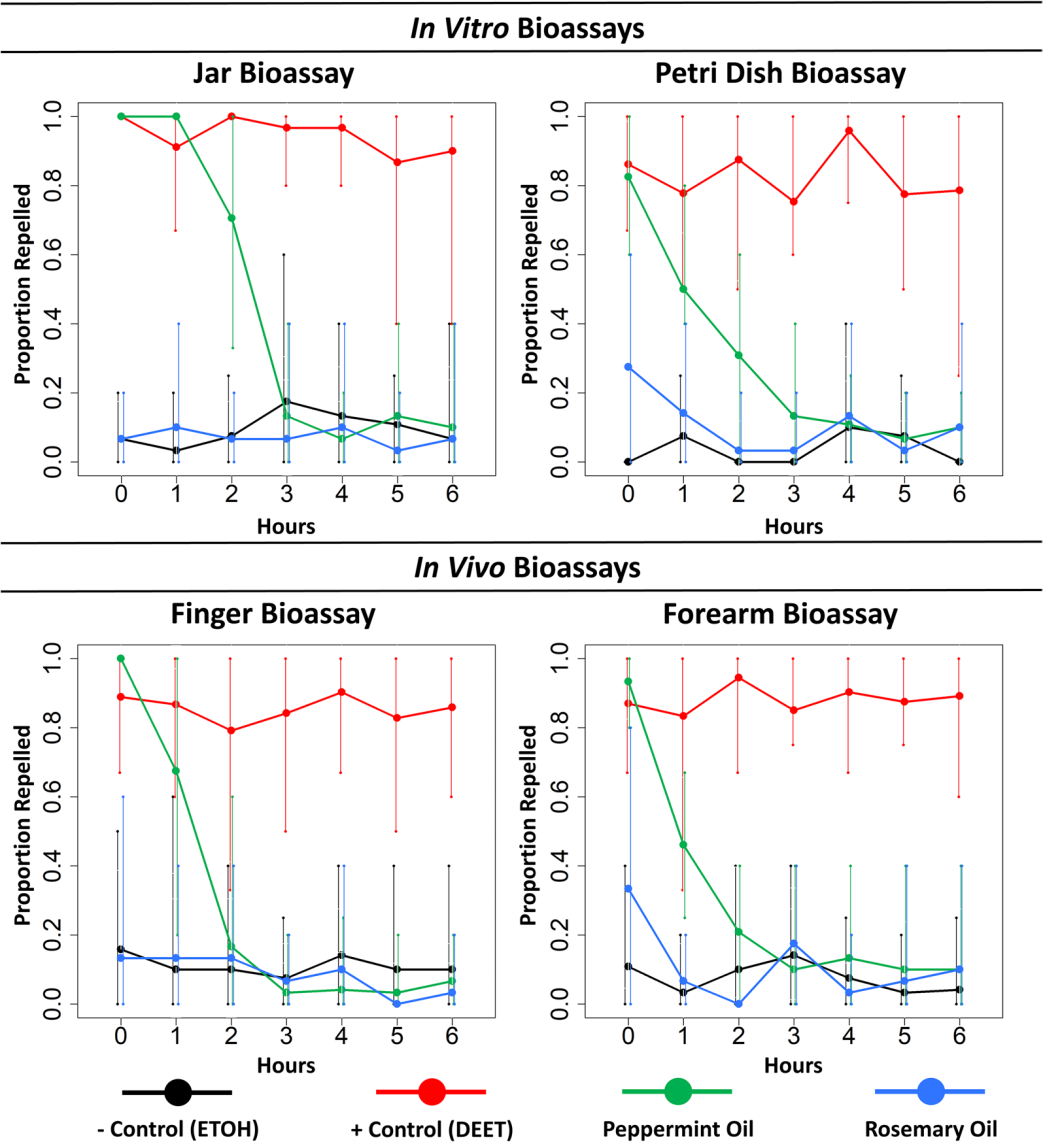
Plots showing the point estimates and range (minimum and maximum) for each time point for the negative control (ETOH) (black), positive control (DEET) (red), peppermint oil (green) and rosemary oil (blue) treatments. These figures only show data for the CDC colony. The proportion repelled for the negative control (ETOH) group was a measure of the portion of nymphs that did not exhibit the expected behavior, rather than true repellency

Using nymphs derived from the CDC colony only, the results did not differ significantly between bioassay methods across treatments or time points after treatment (*df* = 3662, deviance residual = 2.31, *P* = 0.512) (Fig. 3). However, we detected a significant difference between the four treatments (ethanol, DEET, peppermint oil and rosemary oil) (*df* = 1659, deviance residual = 353.3, *P* < 0.001) and between time points after treatment (*df* = 6665, deviance residual = 24.6, *P* < 0.001). In all four bioassays, the DEET treatment remained efficacious for all 6 h of the evaluation period compared with the ethanol treatment (Fig. 3). The percentage of nymphs repelled in the negative control (ethanol) treatment was 8% while in the positive control (DEET) 88% of nymphs were repelled. Across all bioassay methods, the repellent efficacy of rosemary oil did not differ from that of the ethanol treatment, with percentage of nymphs repelled at 9% across time points. Peppermint oil showed some repellent effect for the first hour after treatment but this effect dropped rapidly afterwards, although the oil remained somewhat effective through the second hour in the jar bioassay (Fig. 3). Across bioassays, for peppermint oil, the percentage of ticks repelled was high (93%) at the 0-h time point and fell to 66% at the 1-h time point. The average percentage of nymphs repelled was 14% over the remaining time points across bioassays.

## Discussion

A previous study determined that in vitro and in vivo methods yielded similar effective doses for a number of active ingredients but did not examine whether the duration of repellency varied by method [23]. The findings reported here indicate that when the application rate of repellent active ingredients conforms to EPA guidelines [8] the four bioassay methods tested in our study yield comparable results for the duration of repellency up to 6 h. Most notably, we did not observe significant differences between the in vitro and in vivo methods. Information regarding the comparability of results between bioassay methods improves our ability to compare repellent efficacy information across studies. The lack of effect that we observed when bioassays were conducted on a human subject (i.e. in vivo methods) is encouraging as it means that the results of efficacy testing for new potentially active ingredients, which most commonly are used in vitro methods [6], are likely to carry over to testing on human subjects.

There were a number of minor notable differences between the bioassays. While not statistically significant, the jar bioassay did exaggerate the duration of repellency for peppermint oil slightly, with 100% of nymphs being repelled at the 1-h time point compared with only 63% averaged across the other three bioassays. This likely occurred because ticks were upside-down in the jar bioassay and, therefore, repellent effects may have been slightly enhanced by gravity as nymphs only needed to detach slightly to fall from the top of the container. The in vivo repellency bioassays were conducted on a single subject; little between-subject variation was observed in previous studies that used multiple participants [24, 25]. Aside from these minor caveats, it appears that under controlled conditions, these four bioassay methods yield comparable results.

Due to behavioral differences across tick colonies, we found that the repellency assessment time point needed to be adjusted depending upon the colony that was used. We tested two colonies, one derived from *I.* *scapularis* collected from Connecticut ticks (northern origin) and another derived from Oklahoma ticks (southern origin). Northern and southern clades of *I.* *scapularis* in the USA demonstrate behavioral differences, with ticks from the southern clade tending to quest less actively [16, 26]. We did observe that ticks from Oklahoma were less active and generally took longer to crawl off or through the treated areas. Therefore, the repellency assessment time points tended to be longer for the OSU colony than for the CDC colony. It is possible that this difference is caused by behavioral variation between the northern and southern clades, although differences in rearing conditions between the colonies may have also affected tick behavior. It is also possible that one or both colonies are inbred. While both colonies are intermittently refreshed with adults from either Connecticut and Rhode Island (CDC) or Oklahoma (OSU), it is possible that behavioral differences are due to inbreeding or other selective pressures under colony conditions. This highlights the need to conduct initial testing to ensure bioassay methods are appropriate, not only considering the tick species and life stage, but also the origin and rearing conditions of the colony used.

The use of repellents is generally low in the USA [27–30]. A repellent requiring multiple applications is less likely to be effective, therefore assessing repellency at multiple time points is important. DEET was effective across all 6 h of our study. Peppermint oil initially exhibited similar repellent effects to that of DEET, but the repellent activity dropped rapidly after the first hour. Rosemary oil had a limited repellent effect at all time points, and the results did not differ significantly from those of the negative control (ethanol) treatment. In contrast, a study investigating specific molecular components of rosemary oil found these to be highly efficacious as repellents against the American dog tick, *Dermacentor* *variabilis*, albeit for a short duration [31]. It is therefore worth noting that a lack of repellent activity when testing an essential oil or botanical active ingredient does not necessarily indicate that it contains no compounds that might act as repellents if isolated.

The results of previous studies of ‘25(b) exempt’ active ingredients have varied, depending on the concentration used and tick species targeted [32–34]. Studies for *I.* *scapularis* have primarily focused on finding an effective dose at which ‘25(b) exempt’ active ingredients act as repellents by testing a range of concentrations [11, 19]. A recent study investigated the duration of repellency of 20 different ‘25(b) exempt’ active ingredients at a consistent concentration [12]. Despite using a lower concentration than was used in our study (10% vs 20%), the authors of the study reported similar results, finding that peppermint oil was only equivalently effective to DEET for 44 min and that rosemary oil was not an effective repellent in its raw oil form [12]. The concentration of an active ingredient can affect the duration of repellency in some cases [25], so it is possible that the repellent effects of some ‘25(b) exempt’ active ingredients would last longer at higher concentrations. It is also possible that commercial formulations of these active ingredients may remain effective for a longer time than the unformulated active ingredients we tested due to the presence of chemical stabilizers and synergists, but this possibility requires further evaluation.

## Conclusions

We have demonstrated that different bioassay repellency methods can yield similar results, regardless of whether it is conducted on a human subject. In the present study, we tested four bioassay methods, but our findings cannot be generalized for comparisons between other existing in vitro or in vivo bioassay methods. For example, more complicated methods, such as the moving object bioassay [6, 35] that mimics the warmth and movement of a host, were not evaluated here. We have also shown that it is important to consider the origin of ticks used in repellency bioassays as differences in behavior might affect how repellency is measured. Our data also highlight the need for additional testing for the duration of efficacy of ‘25(b) exempt’ active ingredients. This is particularly important as there are many products currently on the market that utilize ‘25(b) exempt’ active ingredient, whose efficacy is unknown.

## Data Availability

The data that support the findings of this study are available from the corresponding author upon reasonable request.

## Abbreviations

DEET: N,N-Diethyl-meta-toluamide
EPA: US Environmental Protection Agency

## References

[1] Eisen RJ, Eisen L (2018). The blacklegged tick, *Ixodes*
*scapularis*: an increasing public health concern. Trends Parasitol.

[2] Jordan RA, Schulze TL (2020). Availability and nature of commercial tick control services in three Lyme disease endemic states. J Med Entomol.

[3] Eisen L, Stafford KC (2021). Barriers to effective tick management and tick-bite prevention in the United States (Acari: Ixodidae). J Med Entomol.

[4] US Centers for Disease Control and Prevention (CDC). Preventing tick bites. 2023. https://www.cdc.gov/ticks/avoid/on_people.html. Accessed 2 Mar 2023.

[5] US Environmental Protection Agency (EPA). Find the repellent that is right for you. 2023. https://www.epa.gov/insect-repellents/find-repellent-right-you. Accessed 24 Mar 2023.

[6] Adenubi OT, McGaw LJ, Eloff JN, Naidoo V (2018). In vitro bioassays used in evaluating plant extracts for tick repellent and acaricidal properties: a critical review. Vet Parasitol.

[7] Farooq M, Xue RD, Peiper ST, Qualls WA. Evaluating techniques and efficacy of arthropod repellents against ticks. In: Corona C, Debboun M, Coats J, editors. Advances in arthropod repellents. Cambridge: Academic Press; 2022. p. 49–68.

[8] US Environmental Protection Agency (EPA). Product performance test guidelines OPPTS 810.3700: insect repellents to be applied to human skin. Durham: EPA Office of Chemical Safety and Pollution Prevention; 2010. https://downloads.regulations.gov/EPA-HQ-OPPT-2009-0150-0011/content.pdf

[9] US Environmental Protection Agency (EPA). Minimum risk pesticides exempted from FIFRA registration. 2023. https://www.epa.gov/minimum-risk-pesticides. Accessed 2 Mar 2023.

[10] Jordan RA, Schulze TL, Dolan MC (2012). Efficacy of plant-derived and synthetic compounds on clothing as repellents against *Ixodes*
*scapularis* and *Amblyomma*
*americanum* (Acari: Ixodidae). J Med Entomol.

[11] Faraone N, MacPherson S, Hillier NK (2019). Behavioral responses of *Ixodes*
*scapularis* tick to natural products: development of novel repellents. Exp Appl Acarol.

[12] Luker HA, Salas KR, Esmaeili D, Holguin FO, Bendzus-Mendoza H, Hansen IA (2023). Repellent efficacy of 20 essential oils on *Aedes*
*aegypti* mosquitoes and *Ixodes*
*scapularis* ticks in contact-repellency assays. Sci Rep.

[13] Diuk-Wasser MA, Hoen AG, Cislo P, Brinkerhoff R, Hamer SA, Rowland M (2012). Human risk of infection with *Borrelia*
*burgdorferi*, the Lyme disease agent, in eastern United States. Am J Trop Med Hyg.

[14] Mather TN, Nicholson MC, Donnelly EF, Matyas BT (1996). Entomologic index for human risk of Lyme disease. Am J Epidemiol.

[15] Pepin KM, Eisen RJ, Mead PS, Piesman J, Fish D, Hoen AG (2012). Geographic variation in the relationship between human Lyme disease incidence and density of infected host-seeking *Ixodes*
*scapularis* nymphs in the Eastern United States. Am J Trop Med Hyg.

[16] Arsnoe IM, Hickling GJ, Ginsberg HS, McElreath R, Tsao JI (2015). Different populations of blacklegged tick nymphs exhibit differences in questing behavior that have implications for human Lyme disease risk. PLoS ONE.

[17] Schreck CE, Fish D, McGovern TP (1995). Activity of repellents applied to skin for protection against *Amblyomma*
*americanum* and *Ixodes*
*scapularis* ticks (Acari: Ixodidae). J Am Mosq Control Assoc.

[18] Jaenson TG, Pålsson K, Borg-Karlson AK (2005). Evaluation of extracts and oils of tick-repellent plants from Sweden. Med Vet Entomol.

[19] Dietrich G, Dolan MC, Peralta-Cruz J, Schmidt J, Piesman J, Eisen RJ (2006). Repellent activity of fractioned compounds from *Chamaecyparis*
*nootkatensis* essential oil against nymphal *Ixodes*
*scapularis* (Acari: Ixodidae). J Med Entomol.

[20] Bissinger BW, Apperson CS, Sonenshine DE, Watson DW, Roe RM (2009). Efficacy of the new repellent BioUD^®^ against three species of ixodid ticks. Exp Appl Acarol.

[21] R Core Team. 2022. R: a language and environment for statistical computing. Vienna: R Foundation for Statistical Computing. https://www.R-project.org/.

[22] Abbott WS (1925). A method of computing the effectiveness of an insecticide. J Econ Entomol.

[23] Kröber T, Bourquin M, Guerin PM (2013). A standardised in vivo and in vitro test method for evaluating tick repellents. Pestic Biochem Phys.

[24] Semmler M, Abdel-Ghaffar F, Al-Rasheid KA, Mehlhorn H (2011). Comparison of the tick repellent efficacy of chemical and biological products originating from Europe and the USA. Parasitol Res.

[25] Abdel-Ghaffar F, Al-Quraishy S, Mehlhorn H (2015). Length of tick repellency depends on formulation of the repellent compound (icaridin = Saltidin®): tests on *Ixodes*
*persulcatus* and *Ixodes*
*ricinus* placed on hands and clothes. Parasitol Res.

[26] Tietjen M, Esteve-Gasent MD, Li AY, Medina RF (2020). A comparative evaluation of northern and southern *Ixodes*
*scapularis* questing height and hiding behaviour in the USA. Parasitology.

[27] Gould LH, Nelson RS, Griffith KS, Hayes EB, Piesman J, Mead PS (2008). Knowledge, attitudes, and behaviors regarding Lyme disease prevention among Connecticut residents, 1999–2004. Vector Borne Zoonotic Dis.

[28] Hook SA, Nelson CA, Mead PS (2015). US public's experience with ticks and tick-borne diseases: results from national HealthStyles surveys. Ticks Tick Borne Dis.

[29] Niesobecki S, Hansen A, Rutz H, Mehta S, Feldman K, Meek J (2019). Knowledge, attitudes, and behaviors regarding tick-borne disease prevention in endemic areas. Ticks Tick Borne Dis.

[30] Beck A, Bjork J, Biggerstaff BJ, Eisen L, Eisen R, Foster E (2022). Knowledge, attitudes, and behaviors regarding tick-borne disease prevention in Lyme disease-endemic areas of the Upper Midwest, United States. Ticks Tick Borne Dis.

[31] Wong C, Crystal K, Coats J (2021). Three molecules found in rosemary or nutmeg essential oils repel ticks (*Dermacentor*
*variabilis*) more effectively than DEET in a no-human assay. Pest Manag Sci.

[32] Thorsell W, Mikiver A, Tunon H (2006). Repelling properties of some plant materials on the tick *Ixodes*
*ricinus* L. Phytomedicine.

[33] Meng H, Li AY, Costa Junior LM, Castro-Arellano I, Liu J (2016). Evaluation of DEET and eight essential oils for repellency against nymphs of the lone star tick, *Amblyomma*
*americanum* (Acari: Ixodidae). Exp Appl Acarol.

[34] Štefanidesová K, Škultéty Ľ, Sparagano OA, Špitalská E (2017). The repellent efficacy of eleven essential oils against adult Dermacentor reticulatus ticks. Ticks Tick Borne Dis.

[35] Dautel H, Kahl O, Siems K, Oppenrieder M, Müller-Kuhrt L, Hilker M (1999). A novel test system for detection of tick repellents. Entomol Exp Appl.

